# A simple and efficient method for concentration of ocean viruses by chemical flocculation

**DOI:** 10.1111/j.1758-2229.2010.00208.x

**Published:** 2011-04

**Authors:** Seth G John, Carolina B Mendez, Li Deng, Bonnie Poulos, Anne Kathryn M Kauffman, Suzanne Kern, Jennifer Brum, Martin F Polz, Edward A Boyle, Matthew B Sullivan

**Affiliations:** 1 Department of Earth, Atmospheric, and Planetary SciencesCambridge, MA, USA; 2 Division of Geological and Planetary Sciences, California Institute of TechnologyPasadena, CA, USA; 3 Civil Architectural and Environmental Engineering, University of Texas at AustinAustin, TX, USA; 4 Ecology and Evolutionary Biology Department, University of ArizonaTucson, AZ, USA; 5 MIT/Woods Hole Oceanographic Institution Joint Program in Biological Oceanography, Massachusetts Institute of TechnologyCambridge, MA 02139, USA; 6 Department of Biology, Massachusetts Institute of TechnologyCambridge, MA, USA; 7 Civil and Environmental Engineering, Massachusetts Institute of TechnologyCambridge, MA, USA

## Abstract

Ocean viruses alter ecosystems through host mortality, horizontal gene transfer and by facilitating remineralization of limiting nutrients. However, the study of wild viral populations is limited by inefficient and unreliable concentration techniques. Here, we develop a new technique to recover viruses from natural waters using iron-based flocculation and large-pore-size filtration, followed by resuspension of virus-containing precipitates in a pH 6 buffer. Recovered viruses are amenable to gene sequencing, and a variable proportion of phages, depending upon the phage, retain their infectivity when recovered. This Fe-based virus flocculation, filtration and resuspension method (FFR) is efficient (> 90% recovery), reliable, inexpensive and adaptable to many aspects of marine viral ecology and genomics research.

## Introduction

Twenty years since the discovery that viruses are abundant in aquatic systems (Bergh *et al*., 1989; Proctor and Fuhrman, 1990), it is now clear that they are significant ecosystem drivers through their impact on their globally important microbial hosts (Fuhrman, 1999; 2000; Weinbauer and Rassoulzadegan, 2004; Suttle, 2005; 2007; Breitbart *et al*., 2007). For example, viral lysis of cells, which can account for a large percentage of microbial mortality, influences community composition and provides a source of organic substrate through the release of cellular contents. Further, ocean viruses transfer genes from one host cell to another via transduction (Paul, 1999), impacting the evolution of both host and phage. Perhaps most well studied, for example, the cyanobacterial viruses encode and express core photosynthesis genes obtained from their hosts (Lindell *et al*., 2004; 2005; 2007; Millard *et al*., 2004; Clokie *et al*., 2006; Sullivan *et al*., 2006; Bragg and Chisholm, 2008; Hellweger, 2009). Cyanobacterial viruses often contain other genes likely critical in ocean systems, including those involved in scavenging phosphate (Sullivan *et al*., 2005; 2010; Weigele *et al*., 2007; Millard *et al*., 2009) and even nitrogen (Sullivan *et al*., 2010) from seawater.

Investigations of wild viral populations often depend on the concentration of large volumes of water for various assays. On the one hand, less abundant viruses can often be isolated or observed only through the use of concentrated seawater samples (Seeley and Primrose, 1979). On the other hand, the expanding field of viral metagenomics requires large-scale concentrations of seawater (10s to 100s of litres) to obtain enough genetic material for sequencing (e.g. Angly *et al*., 2006). In spite of the importance of research on wild viral populations and their dependence on concentration methods, existing large-scale concentration methods are inefficient, costly and variably reliable.

While it is possible to collect viruses from natural waters using impact filtration onto ≤ 0.02 µm pore-size filters (Steward and Culley, 2010), the low filtration speed and rapid clogging of these filters render this approach only useful for filtering smaller sample volumes (up to a few litres in oligotrophic waters). Several techniques for concentrating aquatic viruses from larger volumes have been developed, including adsorption-elution methods usinglarger pore-size filters (Borrego *et al*., 1991; Katayama *et al*., 2002; Kamata and Suzuki, 2003) and pelleting of viruses with ultracentrifugation (Colombet *et al*., 2007). However, these methods have drawbacks including selective adsorption of viruses to treated filters (Percival *et al*., 2004), limited volume capacity and lack of mobility of ultracentrifugation equipment, and low or variable recoveries of viruses (Fuhrman *et al*., 2005; Colombet *et al*., 2007). These limitations have contributed to an increased usage of ultrafiltration methods to concentrate aquatic viruses, such as vortex flow filtration (VFF) (Paul *et al*., 1991) and subsequently tangential flow filtration (TFF) (Wommack *et al*., 2010).

Tangential flow filtration has been the most prominent method used to concentrate viruses from natural waters because it reduces filter clogging and allows concentration of viruses from the hundreds of litres of sample that are often necessary for genomic and metagenomic analyses of aquatic viral populations (Wommack *et al*., 2010). While TFF is currently the most efficient means of concentrating large volumes of aquatic viruses, it requires expensive equipment (hundreds to thousands of US dollars) and several hours of processing time, and results in highly variable recoveries (2–98%) of viruses (Colombet *et al*., 2007; Schoenfeld *et al*., 2008), depending on factors, such as sample composition, type of TFF used, the amount of backpressure used and the operator's skill in using sample recovery techniques for backflushing of the ultrafiltration membrane. Further, these backflushing procedures render some types (e.g. Helicon spiral TFF cartridges) of these $1000 filters unusable after approximately half a dozen uses (L. Proctor and F. Rohwer, pers. comm.). Considering limitations of the available viral concentration methods, we sought to develop a technique that efficiently and reliably concentrates aquatic viruses and also requires less expensive equipment, requires very little technical expertise, and can be applied under field conditions such as those encountered on oceanographic research cruises.

Here we focus on chemical techniques to develop a virus concentration method suited to marine virus research applications, by adapting flocculation based wastewater treatment techniques. Iron (Chang *et al*., 1958; Manwaring *et al*., 1971; Zhu *et al*., 2005), aluminum (Chang *et al*., 1958; Wallis and Melnick, 1967; Chaudhuri and Engelbrecht, 1970) and polyelectrolytes (Johnson *et al*., 1967) have been used to efficiently flocculate and remove viruses from wastewater (> 99% removal). We explore a flocculation, filtration and resuspension (FFR) method using FeCl_3_ as an efficient, inexpensive and non-toxic flocculent, and the use of biologically benign solvents to redissolve the iron-virus flocculate.

## Results and discussion

### Optimizing a chemistry-based method for recovery of ocean viruses

**Fig. 1:**
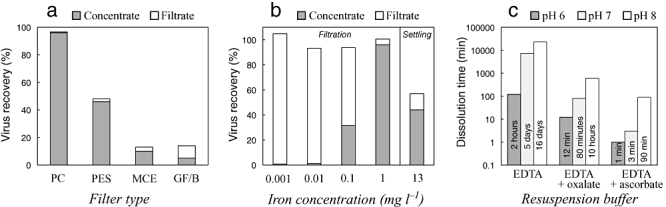
Optimization of virus concentration and redissolution from Biosphere 2 Ocean viral-fraction seawater.A. The effect of various filters on Fe-virus concentrate recovery after flocculation with 1 mg l^−1^ Fe: PC = 0.8 µm polycarbonate filters (Whatman Nuclepore), PES = 0.8 µm polyethersulfone (Pall Supor), MCE = 1.2 µm mixed cellulose ester (Millipore RAWP), and GF/B = 1.0 µm nominal pore size glass fibre filters (Whatman).B. The effect of Fe addition on Fe-virus concentrate recovery by filtration onto a polycarbonate membrane or settling.C. The effect of pH and resuspension buffer on the time required for dissolution of the iron hydroxide flocculate. Resuspension buffers were tested with 0.2 M EDTA in all solutions and the addition of either 0.1 M ascorbate or 0.1 M oxalate to two treatments. One millilitre of buffer was used to dissolve 1 mg of Fe.

As expected, given success with freshwater systems (e.g. Chang *et al*., 1958; Manwaring *et al*., 1971; Zhu *et al*., 2005), FeCl_3_ addition led to efficient virus flocculation with very little virus in the filtrate after the addition of 1 mg Fe l^−1^ to Biosphere 2 Ocean viral-fraction seawater, and effective virus recovery from polycarbonate membrane filters (Fig. 1A). For other membrane materials, the amount of virus in the filtrate was < 10%, suggesting that viruses were minimally lost through the filter and rather were inadequately resuspended off the lower-yielding filter types. Optimal recovery (> 90%) was observed for Fe additions of 1 mg Fe l^−1^ and filtration (Fig. 1B). While settling is possible in a laboratory, it is both impractical on a moving ocean research vessel and inefficient in recovering the Fe-virus precipitate even when larger amounts of Fe (e.g. 13 mg l^−1^) are added (Fig. 1B).

Having successfully collected Fe-virus precipitate onto a polycarbonate filter, we next optimized resuspension methods to maximize recovery off of the filter. To this end, we adapted marine biogeochemistry methods previously used to gently redissolve iron hydroxide precipitates while minimizing harm to phytoplankton cells during nutrient physiology studies (Tovar-Sanchez *et al*., 2003; Tang and Morel, 2006). These techniques use a two-component mixture where the first component promotes dissolution of solid iron hydroxides and a second component chelates the Fe(III) in solution to prevent re-precipitation. Ascorbate (Anderson and Morel, 1982) and oxalate (Tovar-Sanchez *et al*., 2003) have both been used in conjunction with ethylenediaminetetraacetate (EDTA) chelation. Ascorbate promotes iron hydroxide dissolution by reducing seawater-precipitated Fe(III) to seawater-soluble Fe(II), which can then be stabilized with EDTA chelation. Oxalate is thought to promote iron hydroxide dissolution by directly binding and liberating Fe(III) from the surface of iron hydroxide solids, releasing Fe(III) ions into solution where they can be EDTA chelated (Cheah *et al*., 2003; Tang and Morel, 2006). Because EDTA can inactivate viruses by binding magnesium ions (Mg^2+^) (Wells and Sisler, 1969), we provide Mg^2+^ in excess of EDTA's chelating capacity. The dissolution rate of iron hydroxide precipitate was strongly pH dependent regardless of the resuspension buffer used, with dissolution rates at pH 6 roughly two orders of magnitude greater than at pH 8. Both ascorbate- and oxalate-containing buffers acted in a more experimentally practical time frame than EDTA alone (Fig. 1C).

Based on these results, we propose the following new FeFR method for concentration of marine viruses. Seawater may first be pre-filtered (0.22 µm) to remove unicellular algae and other particulate material, depending on the needs of the researcher. One millilitre of a Fe solution (10 g FeCl_3_ l^−1^; Table 1) was added for each 10 l of viral-fraction seawater (final concentration of 1 mg Fe l^−1^ of seawater), gently mixed and incubated 1 h at room temperature to allow Fe-virus flocculate formation. Flocculate can then be collected on a filter (142 mm diameter, 0.8 µm pore-size Whatman polycarbonate membrane filter) minimizing the overpressure (< 15 psi). Filtration time (1–2.5 h per 20 l of seawater) depends upon the sample, and filters should be replaced as needed to maintain flow rate. Place up to three filters into a 50 ml centrifuge tube, and store dark at 4°C until resuspension. Resuspend viruses by adding 10 ml of a resuspension buffer at room temperature (0.25 M ascorbic acid, 0.2 M Mg_2_EDTA, pH 6–7; Table 1), shaking occasionally by hand in order to distribute the buffer over the filters. When the precipitate has dissolved, the virus-containing buffer may be removed for subsequent processing.

**Table 1:** Solution recipes

Concentrated Fe stock (10 g l^−1^ Fe):
4.83 g FeCl_3_•6H_2_O into 100 ml H_2_O
This solution is acidic and should be handled with care.
The solution has expired if a cloudly precipitate forms, do not use. Iron hydroxide precipitate will form quickly if the solution is diluted.
Ascorbate-EDTA buffer:
10 ml 2 M Mg_2_EDTA
10 ml 2.5 M Tris HCl
25 ml 1 M ascorbic acid
Mix components and adjust to pH 6 with ∼1.3 ml 10 M NaOH.
Bring to final volume of 100 ml.
A precipitate may form before the solution pH is adjusted.
Note that solution degrades quickly and should be stored in the dark at 4°C, and used within two days.
Oxalate-EDTA buffer:
10 ml 2 M Mg_2_EDTA
10 ml 2.5 M Tris HCl
25 ml 1 M oxalic acid
Mix components and adjust to pH 6 with ∼4.3 ml 10 M NaOH.
Bring to final volume of 100 ml.
A precipitate may form before the solution is adjusted to pH 6–8.
Modified SM buffer (MSM):
2.33 g NaCl
0.493 g MgSO4•7H_2_O
5 ml 1 M Tris HCl
Mix components into ∼90 ml H_2_O.
Adjusted to pH 7.5 with 10 M NaOH.
Bring to final volume of 100 ml.
Filter sterilize.

### Comparison of the optimized FeCl_3_ method to standard methods

**Fig. 2:**
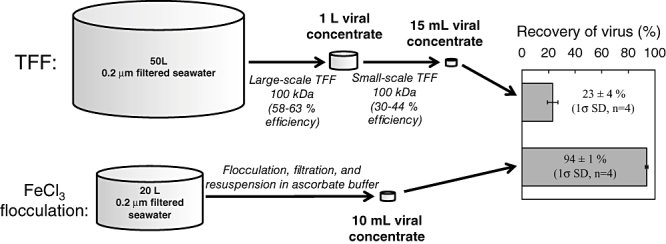
Comparison of viral concentration methods showing the experimental design schematic and resulting concentration efficiency using viral-fraction (< 0.22 µm filtrate) natural seawater from Scripps Pier in San Diego, CA. Recovery is based on virus counts by epifluorescence microscopy.

**Fig. 3:**
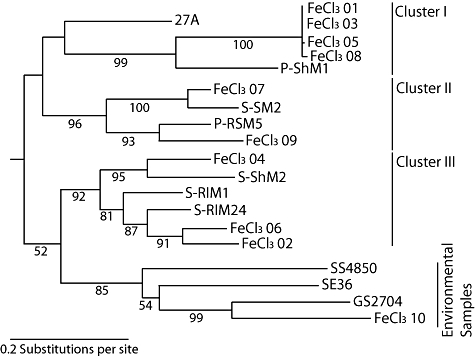
Phylogenetic tree of previously published gene 20 (myovirus portal protein gene) sequences and gene sequences obtained by PCR amplification of gene 20 from Fe-virus concentrates collected at Scripps Pier. Gene sequences obtained in this study are designated as ‘FeCl_3_’.

We compared this new viral concentration method to the standard method (TFF) using viral-fraction seawater from a Pacific Ocean viral community off of Scripps Pier in San Diego, California, USA (Fig. 2, Table 2). Four large-volume samples were concentrated using each method, including three 50 l samples and one 100 l sample by TFF and four 20 l samples by FeCl_3_ flocculation. Average recoveries were 94 ± 1% (1σ SD) for FeCl_3_ and 23 ± 4% (1σ SD) for TFF concentration. To confirm that the Fe-virus concentrates could be used for genetic analysis, we extracted DNA and then amplified, cloned and sequenced myovirus portal protein genes. Even with a small sample size of 10 portal protein gene sequences, the sequences obtained from the Scripps Pier Ocean water sample represented the diversity expected for a wild viral population (Fig. 3).

**Table 2:** Comparison of TFF and FFR viral concentration methods based on a side-by-side testing of these two methods

	TFF	FFR
Set-up cost ($USD)		
Prefiltration (Pump, filter holder, tubing, etc.)	∼$4000	∼$4000
Large-scale TFF	$1603	
Small-scale TFF	$5982	
FFR pump/filter holder		Same as prefiltration
FFR filters		$20
Sample processing		
Volume filtered	50 l	20 l
Time for first viral concentration	1.4 ± 0.4 h (large-scale TFF)	1.4 ± 0.2 h
Time for second viral concentration	4.6 ± 0.8 h (small-scale TFF)	None needed
Time for resuspension from filter	None needed	24 h
Efficiency (% virus recovery)	23 ± 4%	94 ± 1%
Total virus recovery	1.9 × 10^9^ viruses	3.2 × 10^9^ viruses
Final sample volume	15 ml	10 ml

Variability in TFF methodology between labs and modifications for decreasing cost and time of FeCl_3_ flocculation are discussed in the main text.

### Method optimization for flexible sampling needs

The method is robust to many of the variable experimental conditions that might be encountered at sea. First, incubation times of up to 12 h do not affect particle recovery, as evidenced by similar virus recoveries for four separate concentrations over 12 h (Fig. 2). Second, Fe-virus flocculate is amenable to long-term storage either with or without resuspension buffer. After 4 months of storage (dark, 4°C), 85% of virus particles were recovered from Pacific Ocean viral-fraction concentrates (data not shown). This represents only ∼9% loss as compared with the initial 94% recoveries, and this was true regardless of whether the Fe-virus flocculate was resuspended immediately after filtration or 4 months later. Longer vortexing (overnight, dark, 4°C, 100 r.p.m.) of the four-month stored filters increased recovery to 92 ± 3% (1σ SD). Third, processing speeds can be halved for applications where timing is more important than near-complete recovery of viruses. Filtering time for the same Pacific Ocean viral-fraction seawater samples was halved (25 min versus > 1 h for 20 l) by filtering the Fe-virus flocculate though a 0.22 µm pore-size polyethersulfone filter cartridge (Steripak-GP20, Millipore), with a modest drop in recovery to 71–74% (*n* = 2).

**Fig. 4:**
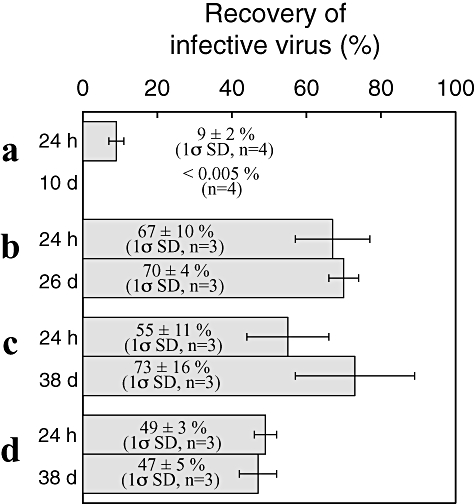
Infectivity of FeCl_3_-flocculated viruses after variable durations of storage (24 h to 38 days) and with different resuspension buffers. Infectivity was assessed by agar overlay plaque assay of flocculated and resuspended virus. Recovery was determined for (A) myovirus resuspended in ascorbate buffer, (B) myovirus resuspended in oxalate buffer and immediately transferred to modified SM buffer for long term storage, (C) myovirus resuspended in oxalate buffer, and (D) siphovirus resuspended in oxalate buffer. Viruses in (A) and (B) were spiked into artificial seawater prior to concentration, while viruses in (C) and (D) were spiked into aged natural seawater prior to concentration.

Beyond SYBR counted viral concentration efficiencies, we optimized Fe-virus concentration with a *Vibrio* phage–host system for use in culture-based studies to maximize the recovery of infective viral particles. Fe-virus concentrate resuspended in ascorbate resulted in low and unstable infective viral recovery (Fig. 4A). These poor infectivity results may be due to damage caused by free radicals formed in ascorbate (Klein, 1945; Murata *et al*., 1986). In contrast, resuspending the Fe-virus concentrate in an oxalate buffer (0.25 M oxalate, 0.2 M Mg_2_EDTA, 0.25 M Tris, pH 6; Table 1) led to efficient and stable infective virus recoveries (47–73% depending upon treatment) for both a myovirus and a siphovirus isolate. Infectivity was maintained through the final time points assayed when viruses were stored in oxalate or exchanged into a standard phage storage buffer (Fig. 4B–D). Additionally, infectivity was tested in a myovirus cyanophage system, S-SM1, with resuspension in either oxalate (13% recovery, *n* = 2) or ascorbate (0%, *n* = 2) buffer, again suggesting the choice of buffer chemistry influences infectivity. Furthermore, oxalate is advantageous because it is more stable at room temperature than buffers made with ascorbate, which must be used within 2 days of preparation (Tovar-Sanchez *et al*., 2003).

Finally, to minimize costs, alternative set-ups might be used. For example, our samples were filtered using a peristaltic pump and a costly stainless steel 142 mm filter holder. Instead, overpressure of a seawater carboy with a home air compressor and a polycarbonate filter holder perform similarly for a total set-up cost of several hundred dollars.

## Conclusions

This Fe-virus concentration method is advantageous in terms of cost, reliability and recovery efficiency. A typical TFF set-up costs over 10 thousand dollars with some costly TFF membranes having a limited lifespan. In contrast, the set-up cost for the FeCl_3_ method can be as little as a few hundred dollars, with minimal per-sample costs. Further, the FeCl_3_ method provides reliable, nearly complete viral recovery (92 to 95%) compared with TFF where recoveries range from 2% to 98% (Colombet *et al*., 2007; Schoenfeld *et al*., 2008), or ∼23 ± 4% as observed here. These improvements are timely given increased sampling throughput requirements to capture temporal and spatial variability, and efforts to develop model systems from lower abundance viral types through culture-based isolations.

## Experimental procedures

Wild ocean viral communities used for optimizing procedures were collected from Scripps Pier, Pacific Ocean (April 2009) and the Biosphere 2 Ocean (May 2009). Whole seawater was pre-filtered through a GF/D membrane (Whatman) in a stainless steel filter holder (Millipore, YY30-142-36) and 0.22 µm Steripak (Millipore GP20), pressured by a peristaltic pump (MasterFlex I/P 77410-10). The ‘viral fraction’ seawater was subsequently concentrated using either large-scale TFF (Amersham Biosciences100 kDa pore-size filter, UFP-100-C-9A) followed by small scale TFF (Millipore Labscale TFF System, XX42LSS11, with Pellicon XL Biomax 100 kDa pore size filter, PXB-100-C-50), or FeCl_3_ flocculation and filtration using the same pump and filter holder as for the initial filtration. Virus concentrations were measured by epifluorescence microscopy after staining with SYBR Gold, according to established procedures (Noble and Fuhrman, 1998).

The suitability of Fe-virus concentrates for genetic analyses was analysed as follows. PCR amplification of T4-like capsid assembly genes (gene 20) was obtained with primer set CPS1.1/CPS8.1 (Sullivan *et al*., 2008) according to the following conditions: initial denaturation step of 94°C for 3 min, followed by 35 cycles of denaturation at 94°C for 15 s, annealing at 35°C for 1 min, ramping at 0.3°C s^−1^, and elongation at 73°C for 1 min with a final elongation step at 73°C for 4 min. The PCR reactions were done in triplicate, pooled into a single tube, purified using a QIAGEN QIAquick PCR Purification kit (Qiagen, Germantown, MD, USA), cloned into a pGEM-T Easy Vector System (Promega, Madison, WI, USA) and 10 clones were then Sanger sequenced at the University of Arizona Genetics Core sequencing centre. The resulting DNA sequences were trimmed to remove PCR primers and ambiguous sequence, and aligned using Clustal X (Gap Opening penalty = 10; Extension = 0.2; DNA matrix IUB) against a suite of published gene 20 sequences chosen to represent the known diversity of these sequences in the wild (Sullivan *et al*., 2008). The alignment was used to calculate a phylogenetic tree using PhyML under the HKY substitution model, with an empirically determined proportion of invariant sites, and transition/transversion ratio (Guindon and Gascuel, 2003).

The recovery of infective viruses from Fe-virus concentrates was tested using vibriophages and a cyanophage. The vibriophages (myovirus Vibriophage 12G01, on *Vibrio alginolyticus* 12G01; siphovirus Vibriophage Jenny 12G5, on *Vibrio splendidus* 12G5) were grown in Difco Marine Broth 2216 and spiked into 500 or 250 ml 0.2-µm-filtered seawater that lacked phages for the assayed *Vibrio* host (Kauffman, data not shown), at final concentrations of ∼10^8^–10^9^ plaque-forming units (PFU) ml^−1^. This mixture was then FeCl_3_ flocculated with 4 mg Fe and filtered onto 47 mm 0.2 µm polycarbonate membranes. Replicate precipitates from separate experiments were resuspended in one of three ways: in an ascorbate buffer, in an oxalate buffer or in an oxalate buffer with subsequent transfer to modified SM (MSM) phage storage buffer (0.4 M NaCl, 0.02 M MgSO_4_, 0.05 M Tris, pH 7.5) (Table 1). Transfer was achieved by centrifugal exchange with multiple rounds of centrifugation (5000 *g*, 20 min, room temperature) in a pre-rinsed 10K Macrosep (Pall) centrifugal device according to manufacturer's instructions, washing with ∼3–4 volumes of MSM. Resuspended samples from all treatments were assayed for infective phage (PFU ml^−1^) by agar overlay plaque assay with glycerol (Adams, 1959; Santos *et al*., 2009). Infectivity was tested 24 h after precipitation and up to 38 days after precipitation and storage in the dark at 4°C.

The cyanophage experiments were done using similar methods, except that the cyanophage (myovirus S-SSM1, on *Synechococcus*) was grown in Pro99 medium (Moore *et al*., 2007) and assayed for titre using the most probable number technique (Sullivan *et al*., 2003).
